# Effect of Ochratoxin A (OTA) on the Immune System: A Systematic Review

**DOI:** 10.3390/toxins17050256

**Published:** 2025-05-20

**Authors:** Yusif Mubarik, Shadrach Tetteh Boyetey, Anastasia Rosebud Aikins, Mohamed Mutocheluh

**Affiliations:** 1 West African Centre for Cell Biology of Infectious Pathogens, University of Ghana, Legon, Greater Accra, Accra P.O. Box LG 54, Ghana; araikins@ug.edu.gh; 2 Department of Biochemistry, Cell and Molecular Biology, School of Biological Sciences, University of Ghana, Legon, Greater Accra, Accra P.O. Box LG 25, Ghana; 3 School of Public Health, Kwame Nkrumah University of Science and Technology, Kumasi P.O. Box 256, Ghana; shadrachtettehboyetey@gmail.com; 4 Department of Clinical Microbiology, School of Medicine and Dentistry, Kwame Nkrumah University of Science and Technology, Kumasi P.O. Box 256, Ghana; mmutocheluh.chs@knust.edu.gh

**Keywords:** ochratoxin A (OTA), innate, adaptive, immunity

## Abstract

Ochratoxin A (OTA) is a mycotoxin with different adverse health effects. The authors conducted a systematic review to evaluate the effects of OTA on the immune system, with more emphasis on its effects on immune system organs, innate and adaptive immunity and related signaling pathways. Studies have demonstrated that exposure to OTA disrupts the functions of immune system organs, resulting in weight loss, histological lesions and a decrease in antibody-secreting cells. There is evidence that OTA impairs epithelial barrier integrity and macrophage function and induces elevated secretion of pro-inflammatory cytokines. In adaptive immunity, OTA regulates T-cell differentiation, particularly Th1 and Th17 subsets, and adversely impacts humoral immunity, ultimately leading to immune suppression.

## 1. Introduction

OTA is among the most harmful common mycotoxins secreted by some *Penicillium* and *Aspergillus* species as a secondary metabolite. OTA-producing fungi survive and multiply at cool, moderate and high climatic temperatures [1]. OTA contamination is found in different geographical and climatic regions. It mostly occurs during the pre-harvest period in cereals like oats, wheat, barley, rice and maize. In addition, the occurrence of OTA in processed beverages, like wine, coffee and grape juice, reveals its high chemical stability during food processing [2,3]. In humans, pigs and calves, OTA exhibits a serum half-life of 35.5 days, 6–10 days and 6 days, respectively [4]. The long half-life in the serum and occurrence in several foods may elucidate the presence of OTA in the blood of more than 70% of individuals examined in Europe and the United States [5,6]. The wide spread contamination of OTA in human biological samples and food products underscores the transnational nature of ochratoxicosis. Studies show OTA levels reaching up to 139.2 μg/kg in different food products across different countries [7,8]. In Indonesia, corn grains and products revealed a mean concentration of 20.385 μg/kg of OTA [9]. Italian and Turkish cereal flour also showed OTA contamination, with ranges from 1.70 to 19.5 μg/kg [10] and 0.12 to 0.59 μg/kg [11], respectively. In addition, maize sampled in Tanzania was contaminated with OTA levels between 16 and 73 μg/kg [12]. Similarly, maize sampled in Nigeria exhibited OTA contamination levels of up to 139.2 μg/kg [7], while about 50% of maize sampled in Ghana exceeded the Ghana Standards Authority (GSA) OTA contamination limits of 5 μg/kg [13]. Furthermore, in Pakistan, 71% of commercially grown maize samples contained OTA levels between 2.14 and 214 μg/kg [14]. Importantly, contamination of OTA in human blood and urine samples from different continents further indicates its transnational impact. In Belgium, 51% of urine sampled in children contained OTA, with levels ranging up to 3.68 ng/mL [15], while in Sierra Leone, OTA was detected in 31% of female and 21% of male children, with concentrations ranging up to 148 ng/mL [16]. Cameroon also showed OTA contamination in 32% of urine samples in children, ranging between 0.04 and 2.4 ng/mL [17]. In support, investigations conducted in European countries indicate the contamination of OTA in adult urine sampled from Sweden (51% prevalence) [18], Belgium (35% prevalence) [15], Spain (0.057–0.562 ng/mL) [19] and Portugal, where contamination up to 100% was reported in some regions [20]. This widespread occurrence of OTA contamination across different geographic regions and its presence in human biological samples and different food products indicate the transnational nature of ochratoxicosis.

OTA contamination in food presents critical economic and health risks due to its widespread occurrence, stability and toxic effects. The metabolism, excretion and toxicokinetics of OTA differ among species, which may influence its impact on different organisms. OTA-induced nephrotoxicity is very common in pigs; hence, pigs serve as an ideal model for studying the effects of OTA exposure on kidney function [21]. In pigs, OTA exposure can result in adverse health effects, such as hyperproteinemia, reduced feeding efficiency, impaired growth, elevated serum creatinine and urea levels and mycotoxic porcine nephropathy [22,23]. Due to the severity of OTA-induced nephropathy, its presence in pig feed is monitored to minimize economic losses [23]. In poultry, OTA exposure causes various adverse health effects, including nephropathy, poor feed conversion and increased susceptibility to infections, leading to reduced productivity [24]. Meanwhile, ruminants exhibit some resistance to OTA toxicity, attributed to the detoxifying effects of rumen micro-organisms [25]. These micro-organisms metabolize OTA into less toxic forms, such as ochratoxin alpha (OTα) [25]. However, dietary changes can impair the rumen’s detoxification capacity, thereby increasing the bioavailability of OTA and its associated adverse health effects in ruminants [26]. Additionally, despite the reduced concentrations of OTA in the milk of ruminants, its contamination in dairy products, such as cheese, has been reported [27]. This highlights the significance of continuous OTA monitoring in animal feeds and dairy products. In humans, OTA exposure primarily occurs through the consumption of OTA-contaminated dairy products, cereals, poultry and pork [28]. However, approximately 60% of OTA exposure in humans can be attributed to the consumption of OTA-contaminated cereals alone [28]. OTA exposure in humans has been associated with renal disorders, such as chronic interstitial nephritis, karyomegalic interstitial nephritis and Balkan endemic nephropathy [28]. Consequently, OTA-induced nephrotoxicity has become a public health concern, particularly due to its ability to mediate oxidative stress via reactive oxygen species (ROS) generation, potentially leading to renal cancer [29]. OTA exposure promotes oxidative stress in kidney cells by inducing ascorbate- and NADH-dependent lipid peroxidation [30]. It then forms a complex with iron (Fe³⁺) before undergoing reduction in the presence of NADH–cytochrome P450 reductase to generate hydroxyl radicals (OH⁻) [30]. These radicals promote membrane lipid peroxidation, resulting in oxidative damage [31]. Lipid peroxidation also impairs cytoplasmic membrane permeability, elevating intracellular calcium (Ca²⁺) levels [31]. This disrupts Ca²⁺ homeostasis, further promoting renal toxicity [31]. Studies have also demonstrated that OTA exposure promotes the activity of the ATP-dependent Ca²⁺ pump in the endoplasmic reticulum (ER) of renal cortex cells, impairing Ca²⁺-mediated cellular functions [32]. The intracellular accumulation of Ca²⁺ promotes oxidative stress by increasing the production of ROS, which in turn impairs renal function by inducing inflammation, cellular damage and apoptosis [33,34]. Additionally, OTA-induced oxidative stress has been shown to impair the Nrf2/ARE pathway by suppressing the activation of Nrf2 and the expression of antioxidant defense genes [35]. In porcine kidney cells (LLC-PK1), OTA exposure suppresses the activity of antioxidant enzymes by inhibiting glutathione peroxidase (GPx) and superoxide dismutase (SOD), while ROS levels increase [35]. Similarly, in rat models and human kidney cells, OTA exposure reduces antioxidant enzyme expression and increases malondialdehyde (MDA) levels [36], indicating OTA-induced oxidative damage. This OTA-induced oxidative stress promotes an inflammatory response by upregulating the expression of NF-κB, intercellular adhesion molecule-1 (ICAM-1) and vascular cell adhesion molecule-1 (VCAM-1) [37], contributing to kidney damage.

Due to the substantial experimental evidence in animals showing that OTA can cause cancer but limited evidence available in humans, the International Agency for Research on Cancer (IARC) has classified this mycotoxin as a Group 2B carcinogen for humans. However, the emerging reports on the role of OTA in oxidative stress, its genotoxicity (including the formation of OTA-DNA adducts) and the involvement of epigenetic factors in OTA-mediated carcinogenesis could potentially lead to it being reclassified as a Group 2A carcinogen (probable human carcinogen), as reviewed by Ostry et al., 2016 [6].

The findings on OTA-related immune responses are mostly contradictory and hard to interpret, which is attributed to differences in models of experiments, routes of administration and dosing regimens [38]. For instance, in in vivo investigations, OTA treatment suppressed antibody secretion, impaired macrophage phagocytosis, induced lymphopenia and elevated the expression of anti-inflammatory cytokines in healthy animals [11,12]. On the contrary, using in vitro experiments, OTA exposure to mononuclear cells and macrophages enhanced the expression of inducible nitric oxide synthase and pro-inflammatory cytokines [38]. Even though these contradictory reports have been attributed to variations in treatment or exposure durations [38,39], further research is needed to clarify the differing immunological outcomes resulting from various routes of administration and dosing regimens, as well as the mechanisms involved. Thus, the immunological effects of OTA are not well understood [40], but studies have shown that it can affect the immune system in various ways [41,42]. Here, we discuss the impacts of OTA on various aspects of the immune system and the mechanisms of these effects.

## 2. Results

### 2.1. The Impact of OTA on the Organs of the Immune System

The immune system is a highly organized system of organs consisting of the primary components, which include the thymus, as well as bone marrow, and the secondary components, such as the Peyer’s patches, spleen, mucosa-associated lymphoid tissues (MALTs) and the lymph nodes, as reviewed in [43,44]. As reviewed in [44], the capacity to initiate a robust immune response relies on the optimal functioning of these organs and the well-coordinated recruitment of immune cells. Consequently, mycotoxins such as OTA that negatively impact the efficient functioning of any immune organ will inevitably have detrimental effects on the overall immune system. Several investigations have detailed the effects of OTA on the immune response in different organisms, and some aspects of these investigations highlight the effects of OTA on key immune system organs. For example, the assessment of histopathology and morphometry from broiler chicks fed either 0.15, 0.3 or 1.0 mg/kg feed contaminated with OTA showed histological damage characterized by pyknotic nuclei, degenerated cells, increased interfollicular connective tissues and empty space in the medullary region of the bursa of Fabricius and the thymus [45]. These effects were dose-dependent, with more severe damage observed at higher OTA levels, particularly at 1.0 mg/kg feed [45]. In support, significant reductions in the weights of the thymus, spleen and bursa of Fabricius were seen in chicks fed a 1.5 mg/kg OTA diet after 21 days [39]. Similarly, the progeny of hens exposed to OTA (3 mg/kg and 5 mg/kg) for 21 days had significantly lower weights and antibody (IgA, IgG and IgM)-producing cells in the bursa of Fabricius compared to non-treated controls [40]. Likewise, the offspring of hens treated with OTA (3 mg/kg and 5 mg/kg) for 14 and 21 days exhibited significantly lower spleen weights due to reduced antibody (IgA, IgG and IgM)-producing cells compared to controls [40]. In contrast, no significant differences in thymus weight were observed in the progeny of hens fed OTA (3 mg/kg and 5 mg/kg) compared to non-treated groups between days 7 and 21 [40]. Additionally, in fishes, low OTA dose (0, 406 or 795 μg/kg) diets did not exhibit any pathological changes [41]. However, higher OTA feed doses (1209 μg/kg of OTA) resulted in a significantly reduced head kidney and spleen weight of the fishes, and this may be attributed to the increased OTA residues in these organs as the OTA concentration in the diet increased from 1200 to 2400 μg/kg [41]. In support, the spleens of the fishes showed signs of disintegrating lymphocytes and reticular cells, with an overall reduction in cell numbers after feeding with a 1209 μg/kg of OTA diet. Apparently, the fish fed with a low OTA concentration (406 or 795 μg/kg) diet did not show any pathological changes in the head kidney after histological examination. However, as observed in the spleen, high OTA concentration (1209 μg/kg to 2406 μg/kg) diets resulted in severe pathological changes, such as disordered lymphatic vessels, a decrease in lymphocytes, congested red blood cells, necrosis of renal parenchymal cells and degeneration of blood vessels in the fish head kidney [41]. In line with the reports above, earlier reports on the mechanism of OTA to induce reductions in the weight of immunological organs suggested that it may be associated with an OTA cytotoxic effect in the lymphoid follicles [42]. In addition, OTA-induced reduced Ig-bearing cell numbers in these organs have been associated with the genotoxic and protein synthesis inhibition effects of OTA [18,19].

### 2.2. Effects of OTA on the Innate Immune Response

#### 2.2.1. The Impact of OTA on Epithelial Barriers

Typically, the gut serves as the immediate point of contact between the host and ingested toxins. The single layer of epithelial cells is linked together by tight and adherens junctions, as well as desmosomes, which form the gastrointestinal mucosa (GIM). The GIM prevents the transfer of toxins and microbes from the lumen of the intestines into systemic sections, but the adverse effect of mycotoxin-mediated GIM impairment has been reported [46]. For example, in ducks, OTA (500 μg/kg body weight) downregulated the expression of tight junction proteins (TJP-1 and occludin) in the jejunum and cecum and disrupted the intestinal microbiota. Similarly, the ingestion of OTA (10 ng) downregulated the tight junction protein Claudin-3 (Cld3) in the small intestine of mice [47]. In addition, mice treated with 250 μg/kg body weight of OTA showed significantly higher diamine oxidase (DAO) activity, D-lactic acid levels and ileal paracellular dextran (FD4) flux, with reduced ileal transepithelial resistance (TER), which are indicators of increased gut permeability and, ultimately, impaired gut barrier function [48].

#### 2.2.2. The Impact of OTA on Macrophages

Macrophages are immune cells with phagocytic capabilities that reside in tissues, originating from various waves of hematopoiesis. They are specialized in recognizing, phagocytosing and eliminating apoptotic cells, harmful micro-organisms and metabolic byproducts, as reviewed in [49]. In porcine alveolar macrophage cell line 3D4/21 (PAMS), short-time (24 h) exposure of OTA (1.0 μg/mL) significantly enhanced cell migration, phagocytic ability, M1 polarization, autophagy and the expression of pro-inflammatory cytokines [38]. However, prolonged (72 h) OTA exposure to PAMS promoted M2 polarization with decreased cell migration, phagocytic ability, autophagy and the expression of pro-inflammatory cytokines [38]. Meanwhile, in lipopolysaccharide (LPS)-pre-stimulated murine macrophages, OTA treatment at concentrations up to 10 ng/mL for 3 days enhanced the release of pro-inflammatory cytokines IL-1β, IL-6, TNF-α and IL-12p40/p70 [47]. Similarly, OTA (0.03 to 1.0 µg OTA/egg) exposure in eggs affected macrophage function in hatched chicks by reducing macrophage phagocytosis and nitrite production in a dose-dependent manner [50]. Furthermore, OTA exposure in breeder hens reduced macrophage phagocytotic activity and nitrite production, with the severity increasing with higher OTA (10.0 OTA mg/kg feed) doses and longer exposure durations (21 days) [51]. Even a low OTA dose (3 mg/kg) in the diet of hens for 7 days reduced both the macrophages’ phagocytic activity and nitrite production of the chicks [40]. Chicks on OTA-contaminated feed (0.1 to 1.5 mg OTA/kg) also showed a significant reduction in the phagocytic activity and nitrite production of abdominal macrophages compared to the non-OTA-treated control group [39]. Thus, prolonged OTA exposure negatively impact the functions of macrophages.

### 2.3. Effects of OTA on the Adaptive Immune Response

#### 2.3.1. The Impact of OTA on T-Cell-Mediated Immunity

T-cells play critical immune roles by preventing diseases and maintaining good health. The stepwise development of T-cells in the thymus generates CD4^+^ and CD8^+^ T-cell subsets. Following stimulation by antigens, naïve T-cells differentiate into memory and effector CD8^+^ and CD4^+^ helper T-cells to mediate immune-modulating function, direct killing and long-term protection [52]. In mice, 10 ng/mL of OTA treatment promoted the differentiation of naive T-cells into Th1 cells, while CD4+ T-cells exposed to supernatants from OTA-treated macrophages displayed increased production of IL-17 [47]. In addition, OTA treatment in mice upregulated the expression of genes critical for Th1 (STAT1 and STAT4) and Th17 (STAT3) cell differentiation in the spleen while downregulating the feedback inhibition mediated by suppressors of cytokine signaling-1 and 3 (SOCS1 and SOCS3) [47]. Furthermore, elevated OTA levels in infants consuming non-breast milk diets correlated with increased activated CD4+ T-cells (characterized by HLA-DR+ and CCR5+ markers) together with elevated CXCL-10 expression, which is associated with mobilization and activation of Th1 cells [53]. Thus, prolonged OTA exposure dysregulate the adaptive immune response.

#### 2.3.2. The Impact of OTA on Humoral Immunity

Humoral immunity is associated with antibody secretion by B cells to protect extracellular spaces by causing the destruction of extracellular micro-organisms and inhibiting the circulation of intracellular infections. B cell activation and differentiation into antibody-secreting cells (plasma cells) are induced by antigens or toxins that normally involve helper T-cells [54]. OTA exposure has been observed to deplete antibody-producing cells in some key immune organs [40], which may obviously affect antibody levels in the OTA-exposed immune milieu. Treatment of laying hens with 250 g/kg of feed of OTA revealed a significant increase in the serum levels of IgA, IgG, IgM and beta-2-microglobulin (2-MG) [55]. On the contrary, in chicks hatched from eggs exposed to OTA (0.01 to 1.0 µg OTA/egg), no significant differences in the total antibody, IgG and IgM titers were observed after 7 days [50]. However, by day 14, the total antibody and IgG titers in chicks hatched from the OTA-exposed eggs (0.5 to 1.0 µg OTA/egg) were significantly reduced (without affecting the IgM levels) compared to the unexposed controls [50]. Additionally, in broilers fed with 1.0 mg OTA/kg, the total antibody titers against sheep red blood cells (SRBC) were significantly reduced compared to the non-OTA-treated controls after 7 days; however, no significant differences in the IgG and IgM levels were observed across all the treated groups [45]. Additionally, after 14 days, the total antibody, IgG and IgM levels showed no significant differences between the OTA-treated birds and the non-treated controls [45]. In support, total antibody and IgG titers in the serum of cockerels fed with OTA (1 to 2 mg/kg of feed) were significantly lower than in unexposed controls at 7 and 14 days post-primary and post-booster SRBC injections [56]. Similarly, the total antibody levels (at days 7 and 14 post-booster injection) and IgG levels (at day 14 post-primary and day 7 post-booster injection) in the serum of chicks fed on an OTA-contaminated diet (0.1 to 1.5 mg OTA/kg diet) were significantly reduced compared to chicks fed a non-OTA-contaminated diet [39]. However, the IgM levels in the OTA-contaminated diet-fed chicks were unaffected at all time points [39].

### 2.4. OTA and Cytokine Secretion

The identification of cytokines as a regulator of immunity has improved our knowledge of the existence of cross-cellular communication during immune responses. In in vitro investigations, 24 h of OTA exposure at 10 ng/mL to nasal epithelial cell cultures (NEC) isolated from both eosinophilic chronic rhinosinusitis (ECRS) patients and healthy individuals (Table 1) showed significantly elevated levels of IL-8 and IL-6 [57]. In addition, in immortalized human microglia-SV40 cells, OTA at 1, 10 and 100 nM dose-dependently increased CXCL8, IL-1β and IL-18 secretion [58]. Similarly, immortalized mouse microglial cells (BV-2) treated with OTA at 500 to 2000 nM for 24 h showed a concentration-dependent rise in IL-6 secretion relative to untreated controls (Table 1) [59]. Furthermore, OTA at 1.0 μg/mL significantly increased TNF-α protein expression, with the lowest level observed at 72 h, and reduced TGF-β expression, which peaked at 72 h in PAMs [38]. Moreover, a 20 µM dose of OTA in human peripheral blood mononuclear cells (hPBMCs) resulted in a peak of IL-6, IL-8 and TNF-α secretion at 12 h (Table 1), which declined by 16 h [60]. However, in human embryonic kidney (HEK293) cells, 1.2 µM OTA treatment significantly reduced the IL-1β levels compared to 0.5 µM and untreated controls [61]. Additionally, while OTA exposure (10 ng/mL) for 72 h did not affect the cytokine levels in unstimulated murine macrophages, a 5–10 ng/mL dose of OTA promoted TNF-α, IL-1β, IL-6 and IL12p40/p70 secretion in pre-stimulated murine macrophages [47]. In addition, OTA treatment of human proximal tubule-derived cells (10 nM) did not alter TNF-α protein release [62]. Similar to most in vitro studies, in in vivo investigations, a 28-day OTA (250 μg/kg) exposure in the diet significantly elevated IL-6, IL-1β and TNF-α levels in the liver and ileum of mice [48]. Additionally, in arthritic mice, OTA exposure at 10 ng/500 mL of saline increased the IL-1β, IL-6 and TNF-α levels [47]. This OTA-mediated arthritis induced increased IFN-γ and IL-17 levels in the splenocytes, with the IL-4 levels unaffected [47]. Furthermore, ducks fed a 235 μg/kg OTA-contaminated diet for 14 days showed significantly elevated serum levels of IL-1β and IL-6 [63]. Similarly, ducklings fed a 500 μg/kg of OTA-contaminated diet for three weeks had significantly increased IL-6 and IL-1β levels in the liver and serum TNF-α [64]. Moreover, a diet containing 250 μg/kg OTA for 28 days elevated the IL-10, TNF-α and IL-2 levels in laying hens, though these changes were not statistically significant [55]. Similarly, in pigs, a 28-day treatment with 250 μg/kg OTA elevated the IL-4 levels in the liver without affecting TNF-α, IFN-γ, IL-1beta, IL-6, IL-8 or IL-10, and had no effect on any of these cytokines in the kidneys [65]. In support, a 30-day OTA treatment (0.05 mg/kg feed) did not alter the IFN-γ, IL-1β, IL-8 or TNF-α levels in the duodenum, kidney or colon but reduced the IL-6 and IL-10 levels in the colon and IL-4 in the duodenum compared to controls in piglets [66]. Thus, prolonged OTA exposure dysregulate cytokine secretion. Table 1 summarizes the effects of OTA on the secretion of cytokines.

### 2.5. Effects of OTA on Signaling Pathways

Cell signaling pathways provide the basis for understanding the underlying mechanisms of function of separate cells, tissues and organs. These pathways provide a simplified model of complex molecular interactions with a living cell. Changes in protein levels and activities in cellular pathways are employed as direct indicators of a response to a particular signal. The effects of OTA on cell signaling pathways in different types of cells in in vivo and in vitro investigations have been reported. For example, prolonged OTA exposure (1.0 μg/mL for 72 h) induced immunosuppression by inhibiting autophagy through the upregulation of p-Akt1 protein expression, which activated the Akt signaling pathway in PAMs [38]. Further, Wang et al. showed that OTA (235 μg/kg body weight) treatment in ducklings for 2 weeks activated the TLR–Myd88 signaling pathway, which resulted in the elevated expression of p-p65 and TLR4-Myd88 mRNA and proteins with an increased p-IKBα/IKBα ratio [63]. Similarly, the TLR4–MyD88 signaling pathway was activated following OTA (500 μg/kg) treatment in the livers of ducks, as shown by the increased protein levels of TLR4, MyD88 and p-p65, as well as the ratio of p-IκBα/IκBα [64]. In addition, compared with untreated control mice, the ileums and livers of OTA-treated mice (250 μg/kg body weight) showed elevated levels of TLR4, MyD88, p-p65 and p65 proteins, confirming that OTA induces elevated pro-inflammatory cytokines through the TLR4–MyD88 and NF-κB signaling pathways [48]. Furthermore, OTA (0.5 µM) exposure for 24 h activated the canonical NF-κB signaling pathway by increasing the expression of p-NF-κB and IKK proteins while simultaneously decreasing the expression of IκBα in HEK293 cells [61]. On the contrary, a 30-day intake of low OTA-contaminated feed (0.05 mg/kg) suppressed the NF-κB signaling pathway by decreasing the expression levels of NF-κB and iNOS genes in the guts of piglets but not in the kidneys [66]. In the p38 mitogen-activated protein kinases (p38 MAPK) pathway, OTA (1209 μg/kg) induced apoptosis in grass carp through both the mitochondrial and death receptor pathways by activating p38-MAPK signaling [41]. Additionally, OTA (50, 250 and 500 nM) activated microglia by activating the extracellular signal-regulated kinase (ERK) and p38–MAPK signaling pathways in mouse microglial cells (BV-2) [59]. In support, OTA (10 nM) exposure for 48 h activated the elevated ERK1/2 pathway by inducing elevated phosphorylation of ERK1/2 but not of c-Jun N-terminal kinase 1/2 (JNK1/2), indicating that ERK1/2 functions as a stimulator mediating OTA-induced pathological changes, whereas JNK1/2 plays an ancillary role in human proximal tubule epithelial-derived cells (HK-2) [62]. Additionally, in chicken heterophils, OTA (5 to 20 µM) treatment for two hours induced the formation of heterophil extracellular traps (HETs) by producing ROS, which is dependent of the activation of NADPH oxidase, ERK and p38–MAPK signaling pathways [67]. To reveal its effect on the Janus kinase/signal transducer and activator of transcription (JAK/STAT) signaling pathway, OTA triggered an elevated inflammatory response by elevating pro-inflammatory and suppressing anti-inflammatory cytokines levels [41]. In addition, treatment of mice with 10 ng/500 mL saline of OTA for 23 days activated the STAT pathways while decreasing the feedback inhibition of suppressor of cytokine signaling (SOCS), marked by a significant elevation in STAT1, STAT3 and STAT4 protein levels, alongside reduced expressions of SOCS1 and SOCS3 [47]. Thus, prolonged OTA exposure dysregulate immune signal response pathways.

## 3. Discussion

Given the critical role of the immune system in protecting against pathogens and preventing tumor development, it is vital to examine how mycotoxins like OTA interfere with immune organs, cells and humoral components. Whether OTA acts as a general cytotoxic or specific immunotoxin agent, its presence may impair endogenous defense systems. OTA immunotoxic effects may involve nonspecific cell destruction by disrupting protein synthesis and important metabolic processes. Additionally, OTA may specifically impair receptor-mediated functions of immune cells, affecting their ability to respond to antigens, as reviewed by Al-anati and Petzinger, 2006 [68]. It is therefore essential to assess whether OTA induces morphological and histological changes in key immune organs, as well as evaluate its impact on immune system functionality.

**Figure 1:**
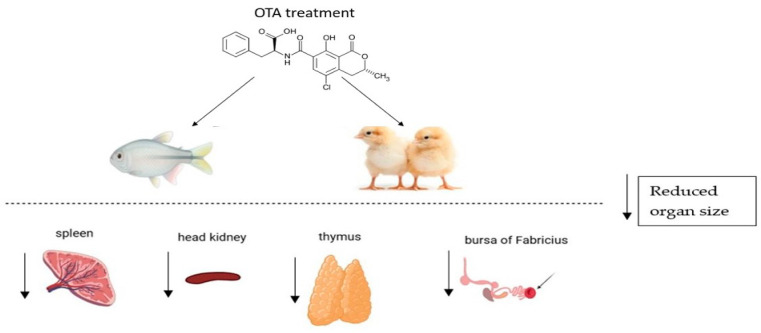
Impacts of OTA exposure on the organs of the immune system. Figure 1 summarizes the effects of OTA on key organs of the immune system, including reducing the size of the thymus, spleen, bursa of Fabricius and head kidney.

**Figure 2:**
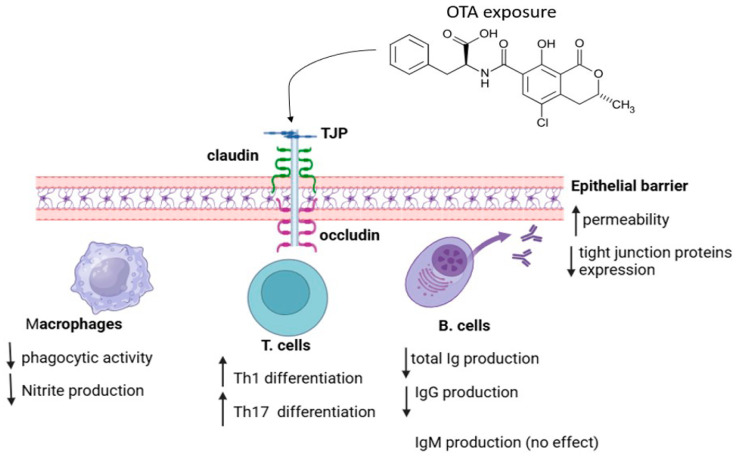
Schematic illustrations of the effects of OTA on epithelial barrier permeability and immune cells. OTA-induced high epithelial barrier permeability, Th1 and Th17 differentiation with reduced macrophage phagocytic activity and nitrite production, total Ig production, IgG production and epithelial tight junction protein expression.

The mechanisms underlying the immune response to OTA exposure or contamination remain incompletely understood due to limited and sometimes contradictory reports. It is important to note that many studies on OTA-mediated immune responses have been conducted on poultry, making it challenging to interpret the findings across different species. In poultry, OTA affects different pathological and physiological parameters, like growth rate, organ weight changes and histopathological alterations in visceral organs [24]. These OTA-induced pathological and physiological effects result in reduced body weight of birds, attributed primarily to decreased feed intake and disruption of protein metabolism [69]. The OTA-mediated suppression of protein metabolism and synthesis in birds is attributed to its competitive inhibition of carboxypeptidase A and linkage to phenylalanine, respectively [70,71]. Exposure to OTA also resulted in increased weights of the kidneys in birds, associated with hyperemia and epithelial enlargement due to efforts to eliminate OTA via the kidney [72,73]. OTA-induced nephrotoxicity is evidenced in birds, resulting in cellular infiltration, tubular atrophy, congested glomeruli, interstitial fibrosis and tubular necrosis [74]. This nephrotoxic effect is dose-dependent, with a higher OTA concentration inducing significant renal damage [75,76]. Conversely, OTA administration resulted in a reduced size of the thymus and bursa of Fabricus, as shown in Figure 1, associated with decreased lymphoid tissue and resulting in immunosuppression [77,78,79]. The bursa of Fabricus undergoes degenerative changes, like reduced cortical diameter and cyst formation in the surface epithelium and interfollicular connective tissue, following OTA exposure [77,78,79]. Additionally, OTA administration may impair the proliferation of lymphoid cells in poultry [77], further contributing to immune suppression, and this has been attributed to OTA-induced impairment of thymus functions [77,78,79]. OTA-mediated thymus impairment may further induce increased pyknotic nuclei, lymphocyte depletion and cortical vacuolation [77,78,79]. These OTA-induced histopathological changes may impair T-cell production. The spleen is also affected by OTA-induced lymphocyte depletion, causing hyperplasia of the splenic parenchyma [77,78,79]. These OTA-induced effects suppress immune responses, making birds more susceptible to infections. Nevertheless, as reviewed in previous studies, OTA-contaminated diets have also been shown to impact immune functions in humans, mice and pigs. Generally, the current review indicates that OTA has an immunosuppressive effect, resulting from histological damage, weight loss and a reduction in antibody-producing cells within key immune organs, like the bursa of Fabricius, thymus, head kidney and spleen, as shown Figure 1 and Figure 2. These adverse effects become more severe at higher OTA concentrations. In support, OTA-mediated suppression of the antibody response resulted in Newcastle disease vaccine failure in chickens [39,40] and increased susceptibility to *E. coli* infections, as seen in turkeys and broilers [80]. It also worsened coccidiosis (*Eimeria tenella*) [81], impaired immunity against *Salmonella* [78] and reduced survival in mice infected with *Pasteurella multocida* [82]. However, whether these OTA-induced immune impairments resulted from either immune cell death mechanism or direct cytotoxicity remains unclear. Both necrosis and apoptosis could contribute to the reduction in antibody-producing cells in lymphoid organs, ultimately leading to lower serum immunoglobulin levels. In addition, autophagy, another programmed cell death mechanism, has been demonstrated to be associated with an OTA-mediated immune suppression mechanism. In an in vitro investigation, OTA inhibited OTA autophagy by upregulating the Akt1 signaling pathway to suppress the immune response in PAMs [38]. As reviewed by Al-anati and Petzinger, this OTA-mediated decline in immune response can be attributed to its genotoxic and protein synthesis-inhibiting effects [68]. Protein synthesis inhibition selectively induces cell death, which ultimately affect immune cell function [83]. Consistently, OTA has been proposed to exert its cytotoxic effects by mimicking phenylalanine [33], resulting in the disruption of critical biochemical processes, particularly in immune function. The structural resemblance of OTA to phenylalanine allows it to act as a toxin mimetic that impairs enzymatic functions [84]. It was hypothesized that OTA directly interferes with phenylalanine-dependent mechanisms by competing with phenylalanine for binding to enzymes involved in protein synthesis, such as phenylalanyl-tRNA synthetase [85]. However, studies have demonstrated that OTA toxicity is not exerted through its phenylalanine component [33]. Rather than competing with phenylalanine, OTA inhibits protein synthesis by disrupting ATP production in mitochondria, leading to reduced ATP availability and, consequently, the inhibition of protein synthesis [33]. ATP depletion results in decreased proliferation of immune cells and impaired antibody production [86], weakening immune responses. OTA-induced immunosuppressive effects are also associated with its ability to suppress mitochondrial respiration by inhibiting carrier proteins in the inner mitochondrial membrane [87], thereby reducing ATP synthesis. Following OTA exposure, elevated migration of lymphocytes to OTA target organs, like the liver and kidneys, occurs [88]. This observation may result in a reduction in immune cell populations in the bloodstream and lymphoid organs, ultimately weakening cell-mediated and humoral immune responses. Besides altering immune cells population in tissues, OTA also impairs their function. For instance, OTA exposure reduces macrophage phagocytic activity and nitrite production, as indicated in Figure 2. Additionally, in chickens, OTA exposure weakened the heterophils’ ability to phagocytose *Enterobacter cloacae* [89]. Similar effects were observed in pigs, where OTA impaired macrophage phagocytosis [48,49]. Additionally, OTA and its metabolite, ochratoxin C, significantly inhibited immune cell proliferation, phagocytic activity, metabolism, nitric oxide synthesis, membrane integrity, division and differentiation [49,50].

**Figure 3:**
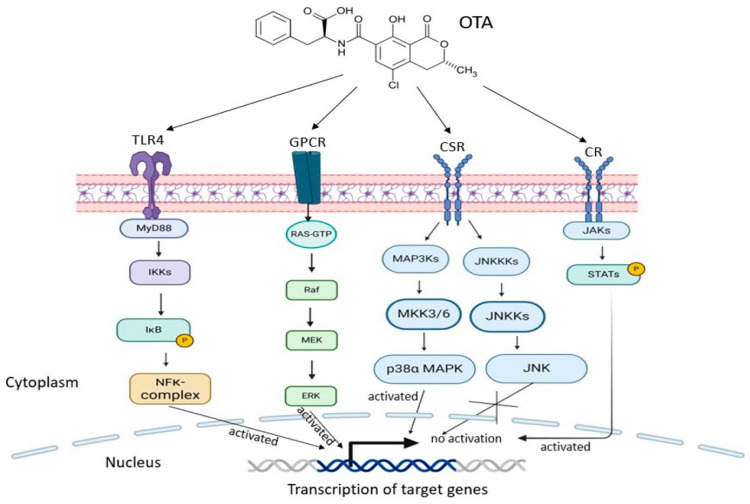
Schematic illustrations of the effects of OTA on the activation of various signaling pathways. OTA exposure induced the activation of JAK/STAT, NF-κB, ERK1/2 and p38–MAPK signaling, but JNK1/2 followed stimulation of cytokines receptors (CR), TLR4, G-protein-coupled receptor (GPCR) and cell surface receptors (CSR), respectively.

Equally significant is OTA’s impact on cytokine secretion, as it may increase the levels of pro-apoptotic cytokines, such as IL-6 and TNF-α, further contributing to immune cell depletion and organ size reduction. Consistently, in both in vitro and in vivo investigations, OTA exposure exerted significant impacts on cytokine production across different cell types, tissue and species, notably by elevating inflammatory cytokines, such as IL-6, IL-8, TNF-α and IL-1β secretion, via the activation of the NF-κB, TLR–Myd88 and JAK/STAT signaling pathways (Figure 3). However, the ability of OTA to induce elevated secretion of pro-inflammatory cytokines has been demonstrated to be time-dependent [38]. OTA (20 µM) treatment of hPBMCs induced elevated production of pro-inflammatory cytokines IL-6, IL-8 and TNF-α at 12 h. However, the levels of these cytokines declined after 16 h of incubation [60]. In addition, OTA (1.0 μg/mL) treatment of PAMs for 24 h to 72 h significantly increased TNF-α protein expression at 24 h, reaching its lowest level at 72 h, while simultaneously decreasing TGF-β expression at 24 h, which peaked at 72 h [38]. To elucidate the underlying mechanism of this paradoxical effect of OTA on inflammation-related cytokine secretion, Su et al. concluded that prolonged exposure to OTA in vitro, rather than short-term exposure, induced immunosuppression and the underlying mechanism involved the inhibition of autophagy via the upregulation of p-Akt1 [38]. Additionally, data from the different studies indicated that OTA exposure enhanced T-cell differentiation into Th1 and Th17 cells [47]. Both Th1 and Th17 cells are known to promote inflammation response. In support, elevated OTA levels in infants has been associated with mobilization and activation of Th1 cells via elevated CXCL-10 expression and activated CD4+ T-cells (characterized by HLA-DR+ and CCR5+ markers) [53]. Remarkably, the heightened CCR5, HLA-DR and CXCL-10 expressions are implicated in infectious disease pathogenesis, including HIV [90], thereby revealing the possibility of OTA to promote HIV mortality and morbidity in infants.

Following its interaction with the intestine, OTA intake has been observed to cause diarrhea, increased translocation of bacteria and rapid inflammation. These effects are indicators of epithelia function disorders. As shown in Figure 2, OTA exposure disrupts the gut barrier function by downregulating tight junction proteins. To demonstrate the direct effect of OTA on epithelial barrier permeability, Maresca et al. reported that OTA treatment of Caco-2-14 and HT-29-D4 cells (human epithelial intestinal cell lines) disrupted microdomains protein contents in plasma membrane. The plasma membrane microdomains proteins regulate the activity of intestinal transport and assembly of tight junction [91]. To elucidate the mechanism underlying this observation, it was shown that OTA treatment modulates Caco-2 cells’ barrier function by removing specific isoforms of claudin [92]. Taken together, these data showed that OTA disrupts the absorption functions and barrier of the intestinal epithelium.

The immunosuppression, immunotoxicity and inflammation effects of OTA across various cell types and species were affirmed by its effects in different cell signaling pathways, as indicated in Figure 3. These findings highlight the complex interactions between OTA and cellular mechanisms, emphasizing its potential effects on human and animal health by adversely modulating signaling pathways, like JAK/STAT, NF-κB, TLR4–MyD88, and p38–MAPK.

## 4. Conclusions

In conclusion, OTA can be characterized as an adverse regulator of inflammation and cellular and humoral immunity with critical implications for animal and human health. OTA exposure and administration significantly impact innate and adaptive immunity by disrupting the functions of immune organs, impairing the permeability of epithelial barriers and compromising macrophage function. Additionally, OTA modulates T-cell differentiation (notably, Th1 and Th17 subsets) and exhibits varied adverse effects on humoral immunity, ultimately leading to immune suppression. Its immunological and cytotoxic effects depend on the concentration and duration of exposure mediated by mechanisms like autophagy, apoptosis, necrosis, inhibition of DNA synthesis and interference with metabolic systems. This highlights the complexity of its role in mediating immune responses.

## 5. Future Directions

### 5.1. Mechanisms of Immune Toxicity

The exact molecular mechanisms of OTA-induced immune suppression are not yet fully understood. Future investigations should focus on identifying key signaling pathways that regulate OTA-mediated immune responses. Additionally, exploring how OTA affects epigenetic modifications and gene expression in immune cells could provide a deeper understanding of its immunotoxic effects.

### 5.2. Acute and Chronic Exposure Effects

Most studies have examined the effects of OTA under controlled laboratory conditions for short durations. However, real-world OTA exposure often occurs at low concentrations and is typically chronic. Further investigations are needed to evaluate the cumulative damage to immune organs from OTA exposure. Longitudinal studies on OTA-mediated immune impairment due to chronic exposure could offer a better understanding of its long-term risks.

### 5.3. Immune Dysfunction and OTA Metabolites

OTA is metabolized in the kidney and liver into metabolites such as ochratoxin C and hydroxylated forms. These metabolites may have different immunotoxic properties. However, almost all of the studies reviewed did not investigate the effects of different OTA metabolites on the various components of the immune system. Further investigations are needed to compare the immunosuppressive effects of OTA and its metabolites on different immune cell types.

### 5.4. Host–Microbiome Interactions and OTA Exposure

The gut microbiome plays crucial roles in immune system function and development. OTA’s ability to disrupt gut barrier permeability was evaluated in some of the studies, but its impact on microbiome–immune system interactions remains underexplored. Future investigations should focus on elucidating how OTA-mediated dysbiosis (microbial imbalance) affects immune responses, particularly in autoimmune diseases, and leads to susceptibility to infections.

### 5.5. Role of Autophagy and Apoptosis in OTA-Induced Immunosuppression

OTA has been demonstrated to inhibit autophagy while promoting apoptosis. Both autophagy and apoptosis are critical programmed cell death mechanisms that regulate immune cell function and survival. However, the exact mechanisms through which OTA exerts these opposing effects remain unclear. Future research should explore how OTA-induced alterations in autophagy and apoptosis affect immune cell proliferation, pathogen clearance and antigen presentation. Identifying key programmed cell-death-related proteins affected by OTA could help develop potential therapeutic targets.

### 5.6. OTA and Vaccine Efficacy

OTA has been demonstrated to impair antibody production in various investigations. Consequently, concerns about its impact on vaccine effectiveness have been raised. Future studies should investigate how OTA exposure influences immune responses to vaccination, particularly in human populations and livestock. Understanding whether OTA compromises vaccine-induced immunity could be crucial for public health strategies.

### 5.7. OTA-Induced Autoimmune and Inflammatory Diseases

Since OTA dysregulates inflammation-related cytokine secretion, it may contribute to the development of autoimmune and inflammatory diseases. Future research should examine whether OTA exposure triggers or exacerbates conditions such as inflammatory bowel disease and lupus. Identifying the association between OTA-induced immune dysfunction and chronic inflammation could aid in developing preventive measures.

### 5.8. Study Limitations

A notable limitation of this review is the heterogeneity of the included articles, which employed different in vivo and in vitro models with varying OTA concentrations and exposure durations. Specifically, the in vivo experiments involved different animal species, such as pigs, mice, chickens, ducks and fishes, each treated with varying OTA concentrations and evaluated across diverse parameters. This variability makes statistical comparisons and meta-analyses of the outcomes impossible. Similarly, the included in vitro studies demonstrated comparable inconsistencies in the experimental designs.

## 6. Materials and Methods

**Figure 4:**
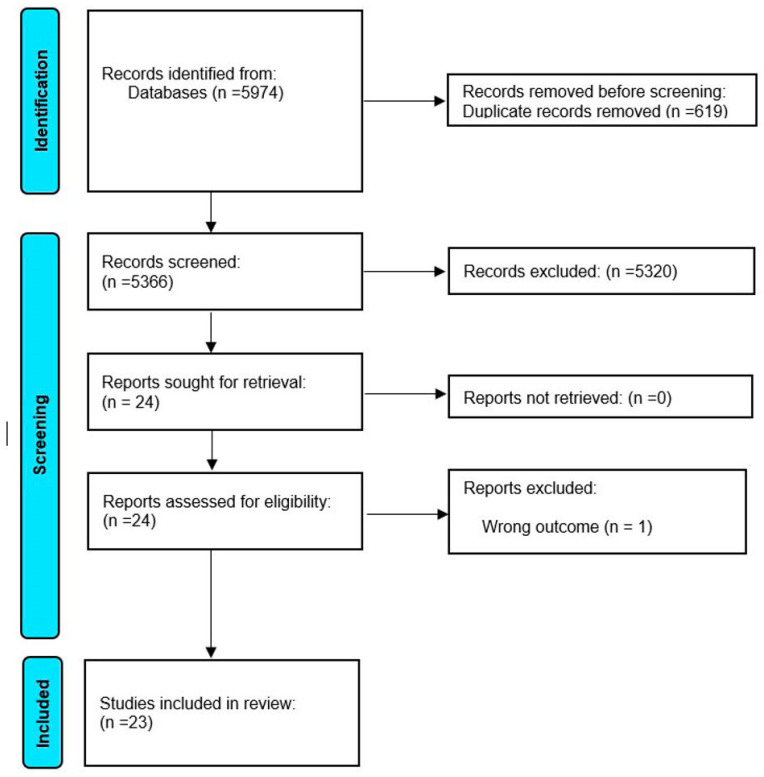
Selection of articles based on the inclusion criteria.

A systematic review of peer-reviewed literature was conducted following the Preferred Reporting Items for Systematic Review and Meta-Analysis Protocol (PRISMA-P, Figure 4). PRISMA-P is a 27-item checklist established to assess bias, enhance the methodological quality and improve the overall rigor of systematic reviews. Currently, no established review protocols exist for this specific systematic review [93]. The final protocol was registered in PROSPERO with identification number CRD42024571369 on 20 July 2024.

### 6.1. Data Sources

We searched MEDLINE (PubMed), EMBASE, Cochrane Library, ScienceDirect (Elsevier), SciELO (Web of Science), gray literature via Google Scholar databases and reference lists from relevant papers. The search was conducted using the keywords mycotoxins, ochratoxin, fungi toxins, immunology, immunotoxicity, immunological, immune response, inflammation and immune for studies published between 2010 and 2024.

### 6.2. Study Selection and Search Strategy

The inclusion criteria of this review were peer-reviewed articles and full reports of studies that investigated the effects of OTA on the various components of the immune system in humans, animals and cell lines between 2010 and 2024. The search strategy used to retrieve articles from databases has been presented in Appendix A (Table A1). Review articles, including systematic, scoping, rapid reviews and meta-analyses, were excluded. In addition, non-English publications and studies with no control group or those that did not examine the effect of OTA alone were excluded. Abstracts of the articles that were retrieved after the literature search were reviewed before obtaining the full articles, where necessary. A summary table of the articles was created, and two authors independently reviewed the papers to guarantee reliability. All disagreements were resolved via discussion before a consensus was reached. The authors reached a consensus regarding the included studies based on the specified inclusion and exclusion criteria. We defined the OTA effect on the immune system as the effect of OTA on

(i) Key organs of the immune system as measured by size, color and functions;
(ii) Innate immune system as measured by cell death, cell recruitment, pro-inflammation or anti-inflammation responses and cytokine secretion;
(iii) Adaptive immune system as measured by cell death, cell differentiation, cell population, antibody secretion and cytokine secretion;
(iv) Drug and vaccine efficacy;
(v) Microbial (bacterial and viral) clearance.

A step-by-step procedure for the literature search is presented in Figure 4. A literature search in NCBI’s PubMed database returned 5325 articles. In ScienceDirect, 618 articles were identified. Additionally, 16 articles were found in Cochrane. Moreover, 15 articles were identified in Google Scholar, bringing the total number of identified articles to 5974. Out of the 5974 retrieved articles, only articles that met the inclusion criteria were included. A total of 619 articles were excluded due to duplication, and 5320 articles were excluded based on information in the abstract and title. A full-text review of the remaining 24 articles led to the exclusion of one study, as it did not specifically evaluate the effects of OTA alone. In all, a total of 23 articles as characterized in Table 2 were included in this study.

### 6.3. Risk of Bias Assessment

In vivo and in vitro studies were assessed using the ToxRtool (Toxicological data Relability Assessment Tool), developed by the European Commission’s Joint Research Centre (JRC) [96]. This tool evaluates study quality based on five key domains: test substance identification, test system characterization, study design description, study results documentation and plausibility of study design and results (Appendix B).

Non-randomized studies included in this review were also assessed using the Risk of Bias in Non-Randomized Studies—of Interventions, Version 2 (ROBINS-I V2) tool [97]. This tool evaluates the potential for bias across seven domains, including confounding, classification of interventions, selection of participants, deviations from intended interventions, missing data, measurement of outcomes and selection of reported results (Appendix B).

Most studies demonstrated high reliability, with in vivo studies scoring between 19 and 20 and in vitro studies ranging from 16 to 18. The primary limitation across the studies was insufficient reporting of physico-chemical properties, but overall, the included studies were deemed methodologically sound for this review.

In [53], an overall moderate risk of bias was determined, primarily due to potential confounding and exposure misclassification, though outcome measurement and reporting were low risk.

## Figures and Tables

**Table 1:** Summary of OTA-mediated cytokine secretion in in vivo and in vitro studies.

Cytokine Secretion Effects	Type of Cells, Tissues or Organs	OTA Concentration	References
Elevated IL-6 and IL-8	Nasal epithelial cells	10 ng/ml	[59]
Elevated CXCL8, IL-1β and IL-18	Human microglia-SV40 cells	(1, 10 and 100) nM	[58]
Elevated IL-6	Mouse microglial cells (BV-2)	(500 to 2000) nM	[59]
Elevated TNF-α	PAMs	1.0 μg/mL	[38]
Elevated IL-6, IL-8 and TNF-α	hPBMCs	20 µM	[60]
Reduced IL-1β	HEK293	1.2 µM	[61]
Elevated TNF-α, IL-1β, IL-6 and IL12p40/p70	Murine macrophages	10 ng/mL	[47]
No effect on TNF-α levels	Human proximal tubule-derived cells	10 nM	[62]
Elevated IL-6, IL-1β and TNF-α	Liver and ileum of mice	250 μg/kg	[48]
Elevated IL-1β, IL-6 levels and TNF-α	Mice plasma	10 ng/500 mL of saline	[47]
Elevated IFN-γ and IL-17	Mice splenocytes	10 ng/500 mL of saline	[47]
Elevated IL-1β and IL-6	Duck serum	235 μg/kg of feed	[63]
Elevated IL-6, IL-1β and TNF-α	Ducklings’ serum and plasma	500 μg/kg of feed	[64]
Elevated TNF-α and IL-2	Hens’ plasma	250 μg/kg of feed	[55]
Did not alter TNF-alpha, IFN-gamma, IL-1beta, IL-6 or IL-8 levels	Pig liver and kidney	250 μg/kg of feed	[65]
Did not alter IFN-γ, IL-1β, IL-8 or TNF-α levels but reduced IL-6 levels	Duodenum, kidney or colon	0.05 mg/kg feed	[66]
Decreased TGF-β	PAMs	1.0 μg/mL	[38]
IL-4 levels not affected	Mice splenocytes	10 ng/500 mL of saline	[47]
Elevated IL-10	Hens’ plasma	250 μg/kg of feed	[55]
Elevated IL-4 with no effect on IL-10 levels	Pig liver	250 μg/kg of feed	[65]
Did not alter IL-4 and IL-10 levels	Pig kidney	250 μg/kg of feed	[65]
Reduced IL-10 and IL-4 levels	Colon or duodenum of piglets	0.05 mg/kg feed	[66]

**Table 2:** Characteristics of included studies.

Reference	Country	Study Type	Sample Size	Participants, Type of Animal and/or Cell	Duration of Exposure	OTA Concentration	Route of Exposure	Outcome
[38]	China	In vitro	N/A	Porcine alveolar macrophage cell line 3D4/21	24, 48 and 72 h	1.0 μg/mL	N/A	Inflammatory response
[39]	Pakistan	In vivo	50	White Leghorn chicks	21 days	0.1, 0.5, 1.0 and 1.5 mg/Kg feed	Oral	Bursa of Fabricius and spleen function effects, phagocytic function and antibody response
[40]	Pakistan	In vivo	36	Breeder hens	21 days	3 and 5 mg OTA/Kg feed	Oral	Bursa of Fabricius, spleen and macrophage function and antibody secreting cells effect
[41]	China	In vivo	21	Grass carp (*Ctenopharyngodon idella*)	60 days	400, 800, 1200, 1600, 2000 and 2400 μg/kg of feed	Oral	Oxidative damage, apoptosis and inflammatory response
[45]	Pakistan	In vivo	30	Broiler chicks	12–42 days	0.15, 0.3 and 1.0 g/kg feed	Oral	Immune toxicity
[48]	China	In vivo	30	Mice	3 weeks	250 μg/kg body weight	Oral	Inflammatory response and mitophagy
[50]	Pakistan	In vivo	120	Chicks	30 days	0.01, 0.03, 0.05, 0.10, 0.50 and 1.00 µg OTA/egg	Oral	Immune suppression
[51]	Pakistan	In vivo	70	Breeder hens	3 weeks	0.1, 0.5, 1.0, 3.0, 5.0 or 10.0 mg OTA/kg feed	Oral	Immune suppression
[53]	South Africa	Human	168	Pregnant women and infants	N/A	N/A	N/A	Immune cells activation
[56]	Pakistan	In vivo	60	White Leghorn cockerels	42 days	1.0 or 2.0 mg/kg feed	Oral	Immune toxicity
[57]	Germany	In vitro	23	Human (nasal epithelial cells)	24 h	10 ng/ml	N/A	Inflammatory response
[58]	USA	In vitro	N/A	Human microglia-SV40	24 h	1, 10 and 100 nM	N/A	Inflammatory response
[59]	Thailand	In vitro	N/A	Murine microglial cells (BV-2)	24 h	50, 250 and 500 nM	N/A	Inflammatory response
[60]	India	In vitro	N/A	Human peripheral blood mononuclear cells	4, 8, 12 and 16 h	20 μM	N/A	Oxidative stress, genotoxicity and inflammatory response
[61]	South Africa	In vitro	N/A	Human embryonic kidney (HEK293) cells	24 h	0.5 mM (sub-IC50), 1.2 mm (IC50) and 2 mm (supra-IC50)	N/A	Inflammatory response and apoptosis
[62]	Germany	In vitro	N/A	Human proximal tubule-derived epithelial cells (HK-2)	48 h	0.3, 1, 10 and 100 nM	N/A	Inflammatory response and fibrosis
[63]	China	In vivo	30	Peking ducklings	14 days	235 μg/kg body weight	Oral	Intestinal microbiota composition and structure alteration, accumulation of LPS and inflammatory response
[64]	China	In vivo	15	Ducks	28 days	235 μg/kg body	Oral	Inflammatory response
[65]	Romania	In vivo	10	Weanling piglets	28 days	250 μg/kg of feed	Oral	Inflammatory response
[66]	Romania	In vivo	12	Piglets	30 days	0.050 mg OTA/kg feed	Oral	Oxidative stress and inflammatory response
[67]	China	In vitro	N/A	Chicken heterophils	2 h	5, 10 and 20 μM	N/A	HET and NET formation
[94]	Spain	In vitro	N/A	Human colon cell line Caco-2 and hepatic cell line HepG2	24 h	5, 15 and 45 μM	N/A	Inflammatory response and cell morphology effect
[95]	Canada	In vitro	N/A	Bovine mammary epithelial cell line (MAC-T)	4, 24 and 48 h	9.6 μmol/L	N/A	Inflammatory response

## Data Availability

No new data were created or analyzed in this study.

## Abbreviations

The following abbreviations are used in this manuscript:

OTA	Ochratoxin A
IARC	International Agency for Research on Cancer
MALTs	Mucosa-associated lymphoid tissues
GIM	Gastrointestinal mucosa
DAO	Diamine oxidase
TER	Transepithelial resistance
LPS	Lipopolysaccharide
SOCS	Suppressors of cytokine signaling
SRBC	Sheep red blood cells
NEC	Nasal epithelial cell cultures
ECRS	Eosinophilic chronic rhinosinusitis
hPBMCs	Human peripheral blood mononuclear cells

## Appendix A. Search Strategy

**Table A1:** Article retrieval search strategy.

Search	Query
1	(((Ochratoxin A[MeSH Terms]) OR (Ochratoxin[MeSH Terms])) OR (Mycotoxins[MeSH Terms])) OR (Fungi toxins[MeSH Terms])
2	((((((Immunology[MeSH Terms]) OR (Immunotoxicity[MeSH Terms])) OR (Immunological[MeSH Terms])) OR (Immune response[MeSH Terms])) OR (Immunity[MeSH Terms])) OR (Inflammation[MeSH Terms])) OR (Immune[MeSH Terms])
3	Filters: Humans, Other Animals, English, from 2010–2024
4	Combination of results 1, 2, linked by Boolean operator “AND”

## Appendix B. Quality Appraisal and Risk of Bias Assessment for Included Studies

**Table A2:** ToxRTool (Toxicological data Reliability Assessment Tool). In vivo studies.

Criterion	[41]	[63]	[64]	[59]	[56]	[45]	[62]	[48]	[50]	[51]	[66]	[47]
Was the test substance identified?	1	1	1	1	1	1	1	1	1	1	1	1
Is the purity of the substance given?	1	0	1	1	1	1	1	1	1	1	1	1
Is information on the source/origin of the substance given?	1	1	1	1	1	1	1	1	1	1	1	1
Is all necessary physico-chemical property information provided?	1	0	1	1	1	1	1	1	1	0	0	0
Is the species given?	1	1	1	1	1	1	1	1	1	1	1	1
Is the sex of the test organism given?	1	1	1	1	1	1	1	1	1	1	1	1
Is information on the strain and other relevant specifications given?	1	1	1	1	1	1	1	1	1	1	1	1
Is age or body weight of the test organisms at the start of the study given?	1	1	1	1	1	1	1	1	1	1	1	1
For repeated dose toxicity studies, are housing and feeding conditions provided?	1	1	1	1	1	1	1	1	1	1	1	1
Is the administration route given?	1	1	1	1	1	1	1	1	1	1	1	1
Are the doses administered or concentrations in application media given?	1	1	1	1	1	1	1	1	1	1	1	1
Are the frequency and duration of exposure, as well as observation time-points explained?	1	1	1	1	1	1	1	1	1	1	1	1
Were negative and positive controls included, where required?	1	1	1	1	1	1	1	1	1	1	1	1
Is the number of animals per group given?	1	1	1	1	1	1	1	1	1	1	1	1
Are sufficient details of the administration scheme provided?	1	1	1	1	1	1	1	1	1	1	1	1
For inhalation/repeated dose studies, were the achieved concentrations verified?	N/A	N/A	N/A	N/A	N/A	N/A	N/A	N/A	N/A	N/A	N/A	N/A
Are the study endpoints and their determination methods described?	1	1	1	1	1	1	1	1	1	1	1	1
Is the description of the study results complete and transparent?	1	1	1	1	1	1	1	1	1	1	1	1
Are statistical methods provided and transparently applied?	1	1	1	1	1	1	1	1	1	1	1	1
Is the study design appropriate for obtaining substance-specific data?	1	1	1	1	1	1	1	1	1	1	1	1
Are the quantitative study results reliable?	1	1	1	1	1	1	1	1	1	1	1	1
Total Score	20	18	20	20	20	20	20	20	20	19	19	19

N/A—Not applicable.

**Table A3:** ToxRTool (Toxicological data Reliability Assessment Tool). In vitro studies.

Criterion	[57]	[94]	[60]	[61]	[58]	[59]	[38]	[95]	[55]	[62]
Was the test substance identified?	1	1	1	1	1	1	1	1	1	1
Is the purity of the substance given?	0	1	1	0	0	1	1	1	1	1
Is information on the source/origin of the substance given?	1	1	1	1	1	1	1	1	1	1
Is all information on the nature and/or physico-chemical properties provided?	0	1	1	0	0	1	1	1	1	1
Is the test system described?	1	1	1	1	1	1	1	1	1	1
Is information on the source/origin of the test system given?	1	1	1	1	1	1	1	1	1	1
Are necessary conditions of cultivation and maintenance given?	1	1	1	1	1	1	1	1	1	1
Is the method of administration given?	1	1	1	1	1	1	1	1	1	1
Are doses/concentrations in application media given?	1	1	1	1	1	1	1	1	1	1
Are the frequency and duration of exposure, as well as observation time-points explained?	1	1	1	1	1	1	1	1	1	1
Were negative and positive controls included, where required?	1	1	1	1	1	1	1	1	1	1
Is the number of replicates or experiment repetitions given?	1	1	1	1	1	1	1	1	1	1
Are the study endpoints and their determination methods described?	1	1	1	1	1	1	1	1	1	1
Is the description of the study results complete and transparent?	1	1	1	1	1	1	1	1	1	1
Are statistical methods provided and transparently applied?	1	1	1	1	1	1	1	1	1	1
Is the study design appropriate for obtaining substance-specific data?	1	1	1	1	1	1	1	1	1	1
Are the quantitative study results reliable?	1	1	1	1	1	1	1	1	1	1
Total Score	16	18	18	16	16	18	18	18	18	18

Each criterion in Table 1 was scored as 1 (criterion met) or 0 (criterion not met). The final reliability classification followed the Klimisch et al. (1997) [98] framework, categorizing studies as Category 1 (reliable without restrictions), Category 2 (reliable with restrictions) or Category 3 (not reliable).

**Table A4:** Risk of Bias in Non-Randomized Studies—of Interventions, Version 2 (ROBINS-I V2) tool.

Domain	[55]
Confounding	Moderate
Classification of Interventions	Moderate
Selection of Participants into the Study	Low
Deviations from Intended Interventions	N/A
Missing Data	Moderate
Measurement of Outcomes	Low
Selection of the Reported Result	Low

N/A—Not applicable. Overall risk of bias: moderate.
